# Evaluations of candidate markers of dihydroartemisinin-piperaquine resistance in *Plasmodium falciparum* isolates from the China–Myanmar, Thailand–Myanmar, and Thailand–Cambodia borders

**DOI:** 10.1186/s13071-022-05239-1

**Published:** 2022-04-12

**Authors:** Run Ye, Yilong Zhang, Dongmei Zhang

**Affiliations:** Department of Tropical Diseases, Faculty of Naval Medicine, Naval Medical University, Shanghai, 200433 People’s Republic of China

**Keywords:** *Plasmodium falciparum*, Dihydroartemisinin-piperaquine, *Plasmepsin*, *pfkelch13*, *pfcrt*

## Abstract

**Background:**

The fast-declining clinical efficacy of dihydroartemisinin-piperaquine (DHA-PPQ) in Cambodia is a warning of the underlying westward dissemination of piperaquine resistance in the Greater Mekong Subregion (GMS). Mutations in the *Plasmodium falciparum* Kelch 13-propeller (PfK13) and the *P. falciparum* chloroquine resistance transporter (PfCRT), as well as *plasmepsin* 2/3 gene amplification, have been discovered as molecular markers for predicting DHA-PPQ treatment failure. Determining whether these genetic variations of *P. falciparum* are linked to DHA-PPQ resistance is critical, especially along the China–Myanmar (CM) border, where PPQ has been utilized for decades.

**Methods:**

A total of 173 *P. falciparum* samples of dried blood spots (DBS) were collected along the CM border between 2007 and 2010, the Thailand–Cambodia (TC) border between 2009 and 2013, and the Thailand–Myanmar (TM) border between 2012 and 2014. PCR and sequencing were used to identified PfCRT mutations, while qPCR was used to determine the copy number of *plasmepsin* 2/3. The prevalence of DHA-PPQ resistance in three locations was investigated using data paired with K13 mutations.

**Results:**

Three fragments of the *pfcrt* gene were amplified for all 173 samples, and seven SNPs were identified (M74I, N75E/D, K76T, H97L, I218F, A220S, I356L). No new PfCRT mutations conferring resistance to PPQ (T93S, H97Y, F145I, M343L, and G353V) were discovered, except for one mutant I218F identified in the TM border (2.27%, 1/44). Additionally, mutant H97L was found in the TC, TM, and CM borders at 3.57% (1/28), 6.82% (3/44), and 1% (1/101), respectively. A substantial K13 C580Y variant prevalence was found in the TC and TM border, accounting for 64.29% (18/28) and 43.18% (19/44), respectively, while only 1% (1/101) was found in the CM border. The K13 F446I variant was only identified and found to reach a high level (28.71%, 29/101) in the CM border. Furthermore, 10.71% (3/28) of TC isolates and 2.27% (1/44) of TM isolates carried more than one copy of *plasmepsin* 2/3 and K13 C580Y variant, while no *plasmepsin* 2/3 amplification was identified in the CM isolates.

**Conclusions:**

Compared with the *P. falciparum* samples collected from the TC and TM borders, fewer parasites carried *plasmepsin* 2/3 amplification and novel PfCRT variants, while more parasites carried predominant K13 mutations at position F446I, in the CM border. Clear evidence of DHA-PPQ resistance associated with candidate markers was not found in this border region suggesting a further evaluation of these markers and continuous surveillance is warranted.

**Graphical Abstract:**

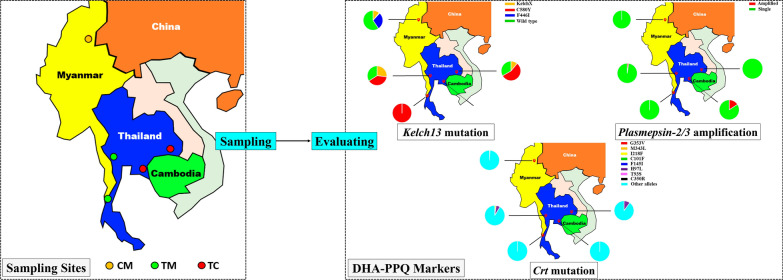

**Supplementary Information:**

The online version contains supplementary material available at 10.1186/s13071-022-05239-1.

## Background

Malaria is a major public health issue that affects the entire world. In 2019, there were still an estimated 229 million malaria cases worldwide [1]. Artemisinin-based combination therapies (ACTs), which combine a fast-acting, rapidly eliminated artemisinin (ART) derivative with a longer-acting partner drug with a longer half-life, have been used as first-line treatment for uncomplicated *Plasmodium falciparum* malaria throughout the world [2] and have exerted significant effects on reducing the incidence of malaria and malaria-related death [3]. Clinical ART resistance, originally identified in western Cambodia [4–6] in 2008, was subsequently observed in other regions of the nation and throughout the Greater Mekong Subregion (GMS) countries [4, 5, 7], owing to the spread and independent emergence of ART-resistant parasites [7–13]. The occurrence and dissemination of ART resistance in *P. falciparum* pose a challenge to the GMS's malaria control and elimination goals. Although the ART resistance increased, the partner drugs of ACTs were capable of eradicating parasites despite the reduced response to ART. However, around a decade after ACTs were introduced, clinical resistance to some widely used ACTs (e.g., artesunate-mefloquine and dihydroartemisinin-piperaquine [DHA-PPQ]) appeared in Cambodia [14–18]. Because of increasing PPQ resistance, the rate of complete therapeutic failure in patients receiving DHA-PPQ has increased significantly in multiple areas in the eastern GMS (e.g., Cambodia, northeastern Thailand, and Vietnam) since 2013 [14, 16, 19–22], necessitating the use of novel ACTs and utilization of triple ACTs [23].

K13 mutations have been identified as molecular markers of partial ART resistance in *P. falciparum* [24]. Within the GMS, substantial geographic variation exists in both the patterns of K13 variants and their prevalence [12, 24–28], indicating diverse drug use histories as well as multiple evolutionary origins of ART-resistant *P. falciparum* [29]. Over 200 non-synonymous K13 variants have been discovered up to now, nine of which (F446I, N458Y, M476I, Y493H, R539T, I543T, P553L, R561H, and C580Y) have been associated with ART resistance [30]. In the eastern GMS, the primary K13 variant is C580Y, whereas, in the western GMS, the most widespread K13 variant is F446I [25, 26, 28, 31]. Additionally, genome-wide association studies (GWAS) demonstrated that copy number amplification of the aspartic proteinase genes *plasmepsin* 2 and *plasmepsin* 3 on chromosome 14 served as a useful gene biomarker for predicting clinical PPQ resistance [32, 33]. Genetic epidemiology studies have revealed that a haplotype involving the K13 C580Y variant (called KEL1) and a haplotype involving a *plasmepsin* 2/3 amplification (called PLA1) have successfully merged to generate a multidrug-resistant co-lineage which has spread throughout the eastern GMS [20, 34]. Consequently, piperaquine resistance emerged in Cambodia mainly developed on a genetic background of K13 C580Y mutation [35]. Intriguingly, with the emergence of PPQ resistance in Cambodia, some novel PfCRT variants (H97Y, F145I, M343L, and G353V) evolved in a Dd2 PfCRT background (74I, 75E, 76 T, 220S, 271E, 326S, 356 T, and 371I) [36], all of which were confirmed via genetic studies as conferring resistance to PPQ [37]. These novel PfCRT variants can serve as molecular markers mediating PPQ resistance.

PPQ was initially utilized as an alternative to chloroquine (CQ) as the first-line therapy for CQ-resistant *P. falciparum* in the 1970s and has been used for a long time in China [38]. Resistance to PPQ was first documented in Yunnan province in the 1990s [39, 40]. Cure rates for a cumulative dose of 25 mg/kg PPQ declined to 33% in the early 1990s [41]. In 2005, China approved the use of DHA-PPQ combination therapy for the treatment of falciparum malaria. Unlike in Cambodia, where the clinical efficacy of DHA-PPQ has been quickly declining, DHA-PPQ was still remarkably effective for treating *P. falciparum* malaria in the China–Myanmar (CM) border, according to recent studies [42, 43].

Hence, in the present study, we evaluated the potential molecular markers related to DHA-PPQ resistance of *P. falciparum* along the CM border, including the novel mutations in the *pfcrt* gene and the *plasmepsin* 2/3 copy number variation (CNV). The K13 variants were also evaluated, as described in our previous study [28]. Additionally, *P. falciparum* isolates collected from the Thailand–Cambodia (TC) and Thailand–Myanmar (TM) borders were also investigated to gain a better understanding of these candidate molecular markers of DHA-PPQ resistance in distinct border areas.

## Methods

### Sample collection

Three *P. falciparum* endemic regions were sampled: Lazan valley (*n* = 101, 2007–2010) along the CM border, Kanchanaburi (*n* = 41, 2012–2014) and Ranong (*n* = 3, 2012–2014) along the TM border, and Trat (*n* = 19, 2009–2013) and Srisaket (*n* = 9, 2009–2013) along the TC border. The isolates were collected from symptomatic malaria patients who were microscopically positive for *P. falciparum*. Dried blood spots (DBS) containing 200–300 μL of peripheral blood were collected on filter paper. Informed consent was acquired from the patients, and all experiments that followed relevant guidelines were approved by the Internal Review Board of Naval Medical University.

### Molecular markers of malaria drug resistance

Genomic DNA was isolated from DBS samples by using the QIAamp DNA Mini Kit (Qiagen, Hilden, Germany). Three *pfcrt* (PF3D7_0709000) fragments (fragment-1*:* 285–854 base pairs [bp], fragment-2: 1080–1379 bp, fragment-3: 2141–2430 bp) were amplified by semi-nested polymerase chain reaction (PCR) to cover potential drug resistance-associated mutations (the expected amplicon sizes are 570 bp, 300 bp, and 290 bp). The primers and PCR conditions are listed in Additional file 1: Table S1a. The PCR products were analyzed by 1.5% agarose gel electrophoresis stained with GoldView (Solarbio, Beijing, China) under UV transillumination. The sequencing reaction proceeded in both directions using an ABI BigDye Terminator Kit (Applied Biosystems, Thermo Fisher Scientific, Waltham, MA, USA). Further analysis was conducted with the assistance of an ABI Prism 3500xL Genetic Analyzer (Applied Biosystems, Thermo Fisher Scientific) in Shanghai (Sangon Biotech).

### Copy number variation assay

Real-time quantitative PCR (qPCR) was utilized to determine the copy numbers of the *pfpm2* (PF3D7 1408000) and *pfpm*3 (PF3D7 1408100) genes on genomic DNA, as previously described [33, 44], and was repeated twice. The *P. falciparum* 3D7 clone was used as a parallel 1 copy control and the *P. falciparum β*-*tubulin* (PF3D7_1008700) gene with a single copy as the internal non-duplicated standard. The primer sequences for *pfpm2*, *pfpm3,* and internal standard of *pfβ*-*tubulin* are shown in Additional file 1: Table S1b. The 2^−ΔΔ*CT*^ method of relative quantification (*C*_*T*_ indicates cycle threshold) was used and adapted to determine the copy numbers of the *plasmepsin* 2 and *plasmepsin* 3 genes, using the formula ΔΔ*C*_*T*_ = (*C*_Target_ − *C*_*T*β_-tubulin) _sample_ − (*C*_Target_ − *C*_*T*β_-tubulin) _3D7_. All reactions were run in triplicate. Copy numbers were considered increased (> 1) when the average of triplicate was above 1.6.

### Data analysis

The multiple sequence alignment of the *pfcrt* gene was conducted in MEGA-X [45], compared with reference sequences (PF3D7_0709000) from the PlasmoDB database (http://www.plasmodb.org). The manual adjustment was conducted by using BioEdit V7.0.9, if required [46]. Gaps were excluded from the analysis and characters were unweighted. Aligned sequences were formatted into a nexus alignment via DnaSP v.5.0 [47], and were used to create a median-joining network in Network 10.2 [48]*.* Fisher’s exact test was performed to assess the difference in the frequency of mutations or CNVs between borders using Statistical Product and Service Solutions (SPSS) software (version 21.0 for Windows). *P*-values < 0.05 were considered statistically significant*.*

## Results

### Prevalence of molecular markers of resistance

**Fig. 1:**
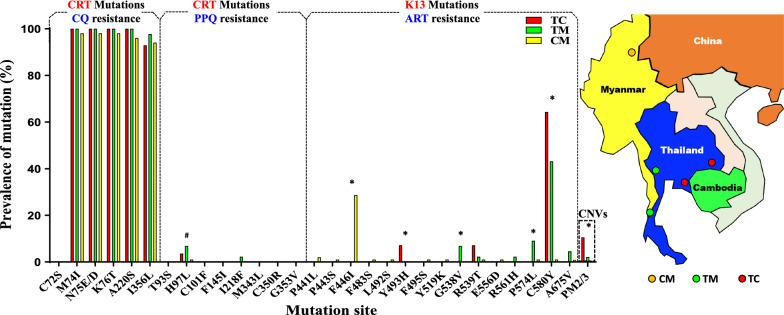
The prevalence of 14 PfCRT mutations, 15 K13 mutations, and CNVs of *plasmepsin* 2/3 in different border regions. CRT mutations associated with CQ and PPQ resistance and K13 mutations associated with ART resistance are displayed in different dashed boxes. *CM* China–Myanmar border (yellow circles), *TM* Thailand–Myanmar border (green circles), *TC* Thailand–Cambodia border (red circles)

To determine the prevalence of molecular markers of drug resistance, a total of 173 *P. falciparum* samples were successfully tested from the three border areas. We first investigated the prevalence of 14 PfCRT mutations including C72S, M74I, N75E/D, K76T, A220S, and I356L conferring resistance to CQ, as well as T93S, H97L, C101F, F145I, I218F, M343L, C350R, and G353V associated with PPQ resistance. A high prevalence of 74I-75E-76T/220S-356L mutations haplotype was observed in the TC, TM, and CM borders, accounting for 92.86% (26/28), 97.73% (43/44), and 93.07% (94/101), respectively. In contrast to previous studies [37, 49, 50], new PfCRT mutations conferring resistance to PPQ (e.g., H97Y, F145I, M343L, I218F, and G353V) were not identified in the current study, except for one (2.27%) of 44 infections harboring the I218F mutation in the TM border region. Additionally, the H97L mutation was detected at a frequency of 3.57% (1/28), 6.82% (3/44), and 1% (1/101) in the TC, TM, and CM borders, respectively (Fig. 1) (Additional file 2: Figure S1, Additional file 3: Table S2, Additional file 4: Table S3).

Following that, the status of *plasmepsin* 2/3 amplification was determined, and real-time PCR analysis revealed that 10.71% (3/28) of TC isolates and 2.27% (1/44) of TM isolates possessed more than one copy of *plasmepsin* 2/3. By contrast, there was no evidence of *plasmepsin* 2/3 amplification in the CM border (Fig. 1) (Additional file 2: Figure S1, Additional file 3: Table S2, Additional file 4: Table S3).

Then, in accordance with our previous study [28], we analyzed the K13 mutations in these isolates collected from the three border areas. The C580Y mutation was identified at a higher prevalence of 64.29% (18/28) and 43.18% (19/44) in the TC and TM borders, respectively, whereas it was identified at a lower prevalence of 1% (1/101) in the CM border. Additionally, the F446I mutation was detected at a high rate of 28.71% (29/101) in the CM border, but not in the TM or TC borders. Notably, the four isolates with *plasmepsin* 2/3 amplifications also carried K13 C580Y mutations, which are known to confer resistance to both DHA and PPQ. Among them, one isolate in the TM border (T67) was observed to carry a PfCRT I218F mutation associated with PPQ resistance (Fig. 1) (Additional file 2: Figure S1, Additional file 3: Table S2, Additional file 4: Table S3).

Fisher’s exact test revealed significant differences in the frequency of specific K13 mutations including F446I, Y493H, G538V, P574L, and C580Y (*P* < 0.05), as well as CNVs of *pm* 2/3 (*P* < 0.05), among the three border regions. In comparison, slight differences were found in only one PfCRT mutation at H97L (*P* = 0.081) (Additional file 3: Table S2).

### Phylogeny

**Fig. 2:**
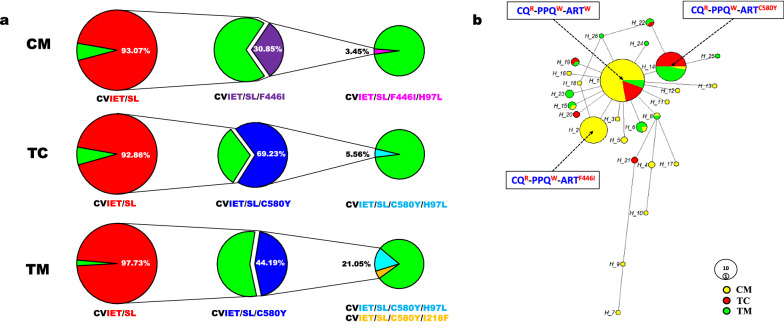
**a** Pie chart of CQ-, ART-, and PPQ-resistant haplotypes and their relationships. CVIET/SL, CQ-resistant haplotype; CVIET/SL/F446I, major ART-resistant type in CM; CVIET/SL/C580Y, major ART-resistant type in TC and TM; CVIET/SL/F446I/H97L, possible PPQ-resistant type in CM; CVIET/SL/C580Y/I218F, CVIET/SL/C580Y/H97L, possible PPQ-resistant type in TC and TM. **b** Haplotype network based on 14 single-nucleotide polymorphisms (SNPs) of *pfcrt* and 15 SNPs of *pfkelch13* in this study. Each observed haplotype is indicated by a filled circle, sized according to its frequency and colored according to the border region represented. Haplotype relationships are indicated by lines; mutational steps between haplotypes are represented by the number of lines. *CM* China–Myanmar border, *TM* Thailand–Myanmar border, *TC* Thailand–Cambodia border, *R* resistant, *W* wild-type

In this study, the chloroquine-resistant haplotype “CVIET/SL” was associated with six PfCRT mutations conferring resistance to CQ. These mutations included C72S, M74I, N75E, K76T, A220S, and I356L (Fig. 2a). Based on this haplotype, the “CVIET/SL/F446I” haplotype with the K13 mutation F446I arose and accounted for 30.85% of the CM population, whereas the “CVIET/SL/C580Y” haplotype with the C580Y mutation arose and accounted for 69.23% of the TC population and 44.19% of the TM population, respectively. Additionally, 3.45% of the haplotype “CVIET/SL/F446I” in the CM population and 5.56% of the “CVIET/SL/C580Y” haplotype in the TC population carried the CRT mutation H97L. In the TC population, 15.79% of the “CVIET/SL/C580Y” haplotype carried H97L, while 5.26% carried I218F (Fig. 2a).

To conduct a thorough comparison of the three border regions, we used 14 SNPs from the *pfcrt* gene and 15 SNPs from the *pfk13* gene to generate 173 new nucleotide sequences (Additional file 5: Table S4). Based on these 173 sequences, a median-joining network was constructed to depict the distribution pattern exhibited by 26 haplotypes (Fig. 2b) (Additional file 6: Table S5). Three major haplotypes were identified among them, namely haplotypes 1, 2, and 14. Haplotype 1 was designated “CQ^R^-PPQ^W^-ART^W^” while haplotype 2 was designated “CQ^R^-PPQ^W^-ART^F446I^”. Haplotype 1 represented the chloroquine-resistant haplotype “CVIET/SL,” while haplotype 2 represented the “CVIET/SL” haplotype with the K13 mutation F446I (CVIET/SL/F446I). In addition, haplotype 14 was designated “CQ^R^-PPQ^W^-ART^C580Y^” to represent the “CVIET/SL” haplotype with the K13 mutation C580Y (CVIET/SL/C580Y). None of the three major haplotypes carried the PfCRT mutations associated with PPQ resistance.

Within haplotype 14, three isolates (collected from the TC border), TC-9, TC-10, and TC-13, carried both the K13 C580Y variant and the *plasmepsin* 2/3 amplification but lacked any PfCRT mutations associated with PPQ resistance. In comparison, haplotype 25 (CVIET/SL/C580Y/I218F) descended from haplotype 14 and was the only one co-lineage to carry the K13 C580Y variant, the *pfpm* 2/3 amplification, and the PfCRT I218F mutation, all of which were associated with DHA-PPQ resistance in the TM border region (Fig. 2b) (Additional file 6: Table S5).

## Discussion

As the GMS strives to eradicate malaria, the intensity of drug selection increases, resulting in rapid adaptation of multidrug-resistant (MDR) parasite populations in the diminishing residual parasites with various genetic backgrounds [24, 51]. This situation provides an unparalleled opportunity to study the evolution of drug resistance, as vital experience regarding the eventual eradication of malaria can be gained. Given that the effectiveness of ACTs is dependent on fast-acting ART derivates, as well as long-acting partner drugs, the resistance to ART may lead to the elimination of a higher proportion of parasites with partner drugs, increasing the risk of partner drug resistance, which may result in clinical failures of ACTs [52].

### Evaluation of candidate markers-K13 variant and *plasmepsin* 2/3 CNVs

In Cambodia, after the introduction of DHA-PPQ, ACT failure rates increased significantly, which was associated with an increase in the prevalence of parasites carrying the K13 C580Y variant and *plasmepsin* 2/3 CNVs [19]. The emergence of the DHA-PPQ resistance may reveal a two-stage selection process in western Cambodia. Initially, the spread of ART resistance resulted in the restriction of parasite genetic diversity, with the majority of parasites harboring K13 variants (e.g., C580Y), and the subsequent emergence of PPQ resistance based on those genetic backgrounds [35, 36]. Following that, parasite lineages carrying the K13 C580Y variant and *plasmepsin* 2/3 CNVs spread quickly throughout eastern GMS within a short period, resembling a hard selective sweep [20, 53]. The present study discovered a high prevalence of the C580Y mutation along the TC and TM borders, which increased significantly (33.33% in 2009–2010, 55.56% in 2011–2012, and 84.62% in 2013) along the TC border, near northwest Cambodia (Additional file 7: Figure S2a). Four of these C580Y mutation isolates contained multiple copies of *plasmepsin* 2/3; three were discovered on the TC border (23.08% in 2013), while one was discovered on the TM border (5.88% in 2013) (Additional file 7: Figure S2b). These findings corroborated previous retrospective genetic studies conducted in Southeast Asia [34, 53]. Though *plasmepsin* 2/3 copy number was previously reported to be associated with PPQ resistance and served as a helpful molecular marker for predicting DHA-PPQ clinical failures in eastern GMS [20, 22, 34, 54], genetic inactivation and overexpression of *plasmepsin* 2/3 genes in the 3D7 background produced inconsistent results [55, 56]. Disrupting *plasmepsin* 2/3 increased parasite sensitivity to PPQ but not to other antimalarials [55], whereas overexpression of these enzymes had no effect on parasite susceptibility to PPQ, CQ, and artesunate [56]. Thus, the role of *plasmepsin* 2/3 copy number in PPQ resistance may be genetically determined [52].

*Plasmodium falciparum* isolates from the CM borders possessed the predominant K13 F446I mutation, which was similar to that found in parasites from northern Myanmar [25, 26]. According to Zhang’s study, the frequency of F446I variant increased gradually from 2007 to 2013 [57], reaching 17.6% in 2007, 21.1% in 2008, 7.1% in 2009, 33.3% in 2010–2012, and 62.5% in 2013. Similarly, in the current study, the prevalence of the C580Y mutation was as low as 1% (1/101) in the CM border. In comparison, this region had a higher prevalence of F446I (28.71%), which increased significantly from 2007 to 2010 (15.79% in 2007, 25% in 2008, 40.00% in 2009, and 36.40% in 2010) (Additional file 3: Tables S2) (Additional file 7: Figure S2a). Inserting the F446I variant into K13 resulted in an increase in ring survival rates detected using RSA_0–3 h_, indicating that this variant might be associated with ART resistance [58]. Additionally, this variant was related to increased PPQ IC50, IC90, and AUC results in contrast to the wild-type (WT) parasites, implying that F446I may represent the K13 background mutations in the CM border region where PPQ resistance develops [52], similar to the C580Y mutation in Cambodia. Si et al. discovered that 5.8% of the 120 parasites obtained within the CM borders had PSA (PPQ survival assay) values greater than 10% (survival rate cutoff), confirming the parasites' PPQ resistance in this area. However, similar to the findings in this paper, no *plasmepsin* 2/3 amplification was detected (Additional file 7: Figure S2b), casting doubt on the utility of *plasmepsin* 2/3 CNVs for PPQ resistance monitoring beyond the eastern GMS [52].

### Evaluation of candidate markers-novel PfCRT variants

Population genomics studies demonstrated that the DHA-PPQ-resistant *P. falciparum* KEL1/PLA1 co-lineage with the K13 C580Y variant and *plasmepsin* 2/3 amplification originated in western Cambodia and spread rapidly in eastern GMS [20, 53]. Numerous subgroups of KEL1/PLA1 co-lineage background parasites have developed new PfCRT mutations [53]. These new PfCRT variants (T93S, H97Y, F145I, I218F, M343L, and G353V) were identified in parasites collected from Cambodia [36, 37, 50], with genetic evidence revealing that they are capable of mediating DHA-PPQ resistance without *plasmepsin* 2/3 amplification [37]. In the present study, only one isolate with the K13 C580Y variant and *plasmepsin* 2/3 amplification from the TM border (in 2013) carried the PfCRT I218F mutation (Hap 25), which shared genetic characteristics with the DHA-PPQ-resistant isolates from eastern GMS (Additional file 7: Figure S2c). The absence of new PfCRT mutations could be explained by the short time span used to collect samples in this study, as new PfCRT mutations became more prevalent in 2016–2017 [53]. Interestingly, five isolates with the PfCRT H97L mutation have been identified, three from the TC borders (in 2014) based on the “CVIET/SL/C580Y” haplotype (Hap 22), one from the CM border (in 2010) based on the “CVIET/SL/F446I” haplotype (Hap 18), and one from the TM border (in 2011–2012) based on the “CVIET/SL” haplotype (Hap 26) (Additional file 7: Figure S2c). Nevertheless, none of these isolates harbored more than one copy of *plasmepsin* 2/3. According to Si’s study, no novel PfCRT variants (H97Y, F145I, M343L, or G353V) were detected in *P. falciparum* isolates from the CM border region. However, they discovered that one isolate harbored the H97L variant from the CM border exhibiting a significantly increased CQ IC_50_ value, as well as increased PPQ IC_50_, IC_90_, PSA, AUC values, in comparison to the parasite harboring the Dd2 haplotype *pfcrt* [52]. Nonetheless, this variant was detected in only one parasite in Cambodia during an ACT efficacy study, and it was not associated with decreased PPQ sensitivity [50]. Additional research is needed to determine whether the PfCRT H97L mutation is associated with PPQ resistance.

Previous studies conducted in the CM border revealed that DHA-PPQ was still remarkably effective in treating falciparum malaria between 2007 and 2013 and that the sensitivity of *P. falciparum* to DHA-PPQ had not changed significantly in this area [42, 43]. Coinciding with the established sustained clinical efficacy of DHA-PPQ in the CM border area, Si’s study confirmed that clear evidence of PPQ resistance in association with these molecular markers was not found in isolates collected from this region between 2007 to 2016 [52]. Additionally, the fluctuating susceptibility to PPQ over time indicates that resistance to PPQ occurred one or more times but did not spread widely. It would be intriguing to determine if this parasite population evolved as a result of strong selection via frequent and long-term use of DHA-PPQ in this border region or due to parasites dissemination from other regions within the GMS.

## Conclusion

The present work evaluated candidate DHA-PPQ resistance biomarkers in *P. falciparum* isolates from the CM, TM, and TC borders using the *pfkelch13* and *pfcrt* genes, as well as *plasmepsin* 2/3 CNVs. In comparison to *P. falciparum* parasites collected from the TC and TM borders, fewer *P. falciparum* parasites possessed *plasmepsin* 2/3 amplification and novel PfCRT variants, while more samples carried predominant K13 mutations at position F446I, along the CM border. Because of a lack of phenotypic or DHA-PPQ susceptibility data, clear evidence of DHA-PPQ resistance associated with these candidate markers was not found in parasites from the CM border region. Further evaluation of these markers and continuous surveillance is warranted.

## Supplementary Information

**Additional file 1: Table S1**. (**a**) The primer sequences and PCR conditions of three fragments of *pfcrt*. (**b**) The primer sequences for pm 2, pm 3, and internal standard of *P. falciparum* β-tubulin were used in qPCR.

**Additional file 7: Figure S1**. The K13 mutation status, *plasmepsin* 2/3 amplification status, and PfCRT mutation status by site and country. (**a**) KelchX mutation status indicates parasites with a K13 mutation other than C580Y and F446I. (**b**) “Single copy” indicates parasites without amplification of *plasmepsin* 2/3. (**c**) Other (PfCRT) alleles indicate parasites carrying no mutations at positions 93, 97, 145, 218, 343, 350, and 353 of the *pfcrt* gene. The shapefile map of Southwestern China, Cambodia, Myanmar, and other countries was downloaded and prepared by using Pixelmap Generator-Beta online (amCharts, Vilnius, Lithuania) (https://pixelmap.amcharts.com/), which is copyright free.

**Additional file 3: Table S2**. The prevalence of K13 mutations, *plasmepsin* 2/3 CNVs, and PfCRT mutations in different border regions. Fisher’s exact test was done to assess the difference in the frequency of mutations or CNVs between borders using SPSS (version 21.0 for Windows). *P* < 0.05 was considered statistically significant.

**Additional file 4: Table S3**. Detailed information of PfCRT mutations, *plasmepsin* 2/3 amplifications, and K13 mutations of 173 *P. falciparum* which were collected from different border regions.

**Additional file 5: Table S4**. SNPs of *pfcrt* and pfkelch13 were used for building a haplotype network of 173 *P. falciparum* collected from different border regions.

**Additional file 6: Table S5**. Detailed information of 26 haplotypes was generated using 173 SNP sequences based on PfCRT and K13 mutations. Cells with different colors denote the K13 or PfCRT mutations that were detected within the haplotypes, as well as the amplification of *plasmepsin* 2/3. Sample IDs in bold and marked with an asterisk denote the parasite carried the amplification of *plasmepsin* 2/3 within haplotype 14.

**Additional file 7: Figure S2**. (**a**) Frequency of K13 mutations in CM, TC, and TM in different year groups. KelchX mutation status indicates parasites with a K13 mutation other than C580Y and F446I. (**b**) Frequency of *plasmepsin* 2/3 amplifications in CM, TC, and TM in different year groups. ”Single copy” indicates parasites without amplification of *plasmepsin* 2/3. (**c**) Frequency of PfCRT mutations in CM, TC, and TM in different year groups. ”Single copy” indicates parasites without amplification of *plasmepsin* 2/3. Other (PfCRT) alleles indicate parasites carrying no mutations at positions 93, 97, 145, 218, 343, 350, and 353 of the *pfcrt* gene.

## Data Availability

Data supporting the conclusions of this article are included within the article and its additional files. The datasets generated and/or analyzed in the current study are available in GenBank (http://www.ncbi.nlm.nih.gov/). The nucleotide sequence data reported in this paper have been deposited in the GenBank DNA database (https://www.ncbi.nlm.nih.gov/genbank/) with accession number OM945736-OM945807.

## Abbreviations

DHA-PPQ: Dihydroartemisinin-piperaquine
ART: Artemisinin
CQ: Chloroquine
ACTs: Artemisinin-based combination therapies
GMS: Greater Mekong Subregion
ATS-MFQ: Artesunate-mefloquine
K13: Kelch 13-propeller
PfCRT: *Plasmodium falciparum* chloroquine resistance transporter
DBS: Dried blood spots
CM: China–Myanmar
TM: Thailand–Myanmar
TC: Thailand–Cambodia
GWAS: Genome-wide association
MDR: Multidrug resistance
CNV: Copy number variation
PSA: PPQ survival assay
AUC: Area under the curve
