# Supplementary material for: Evaluations of candidate markers of dihydroartemisinin-piperaquine resistance in Plasmodium falciparum isolates from the China–Myanmar, Thailand–Myanmar, and Thailand–Cambodia borders
Source: Parasit Vectors. 2022 Apr 12;15:130. doi: 10.1186/s13071-022-05239-1 (PMC9004172; doi:10.1186/s13071-022-05239-1)
Supplement: Supplementary file 1 — Additional file 1: Table S1. (a) The primer sequences and PCR conditions of three fragments of pfcrt. (b) The primer sequences for pm 2, pm 3, and internal standard of P. falciparum β-tubulin were used in qPCR. [file 13071_2022_5239_MOESM1_ESM.docx]

**Table S1a. The primer sequences and PCR conditions of three fragments of pfcrt**

| **Fragments** | **Sequences (5’→3’)** | **Cycling conditions** | **Product size (bp)** |
| --- | --- | --- | --- |
| ***Pfcrt-1*** | F1: TGGAGGTTCTTGTCTTGGTAAATGT (1^st^, 2^nd^)  R1: AAGCAGAAGAACATATTAATAGGAA (1^st^)  R2: AATAGGAATACTTAATTGAAGAACA (2^nd^) | 94°C 5 min,  [98 °C 20 s, 56 °C 30 s, 68 °C 30s] ×30 cycles,  68 °C 5 min | 570 |
| ***Pfcrt-2*** | F1: TCTCGGAGCAGTTATTATTGTTG (1^st^, 2^nd^)  R1: ACTATTTCCCTTGTCATGTTTG (1^st^)  R2: ATTTCCCTTGTCATGTTTGAA (2^nd^) |  | 300 |
| ***Pfcrt-3*** | F1: CGCATTGTTTTCCTTCTTTAAC (1^st^)  F2: ACATTTGTGATAATTTAATAACCAGC (2^nd^)  R1: TATATCTTTTTAATTCTTACGGCTA (1^st^, 2^nd^) |  | 290 |

**Table S1b. The primer sequences for pm2, pm3, and internal standard of *P. falciparum* β-tubulin**

| **Primer sequence** | **Sequences** (5’→3’) | **Product size (bp)** |
| --- | --- | --- |
| *Pfpm2*_CN_F ^a^ | TGGTGATGCAGAAGTTGGAG | 79 |
| *Pfpm2*_CN _R ^a^ | TGGGACCCATAAATTAGCAGA |  |
| *Pfβ*-*tubulin*_CN_F ^b^ | TGATGTGCGCAAGTGATCC | 79 |
| *Pfβ*-*tubulin*_CN_R ^b^ | TCCTTTGTGGACATTCTTCCTC |  |
| *Pfpm3*_CN_F ^a^ | GGTAGTGAGTTTGATAATGTGG | 160 |
| *Pfpm3*_CN _R ^a^ | CACAAGACTCTGATGTACA |  |

Note, 'a' denotes primer sequences adapted from reference 18; 'b' denotes primer sequences adapted from reference 13.
