# Supplementary figures and images for: Evaluations of candidate markers of dihydroartemisinin-piperaquine resistance in Plasmodium falciparum isolates from the China–Myanmar, Thailand–Myanmar, and Thailand–Cambodia borders
Source: Parasit Vectors. 2022 Apr 12;15:130. doi: 10.1186/s13071-022-05239-1 (PMC9004172; doi:10.1186/s13071-022-05239-1)

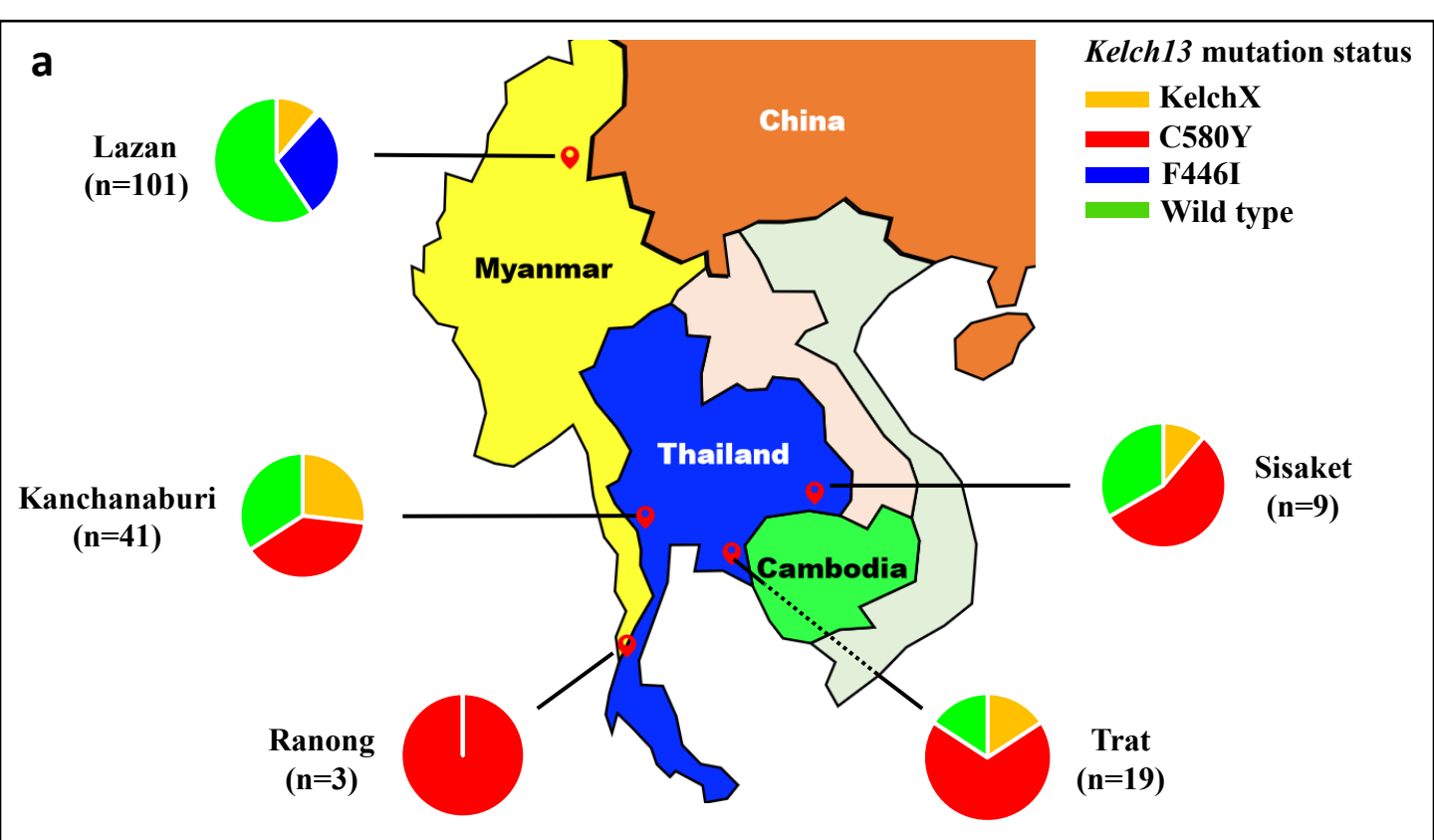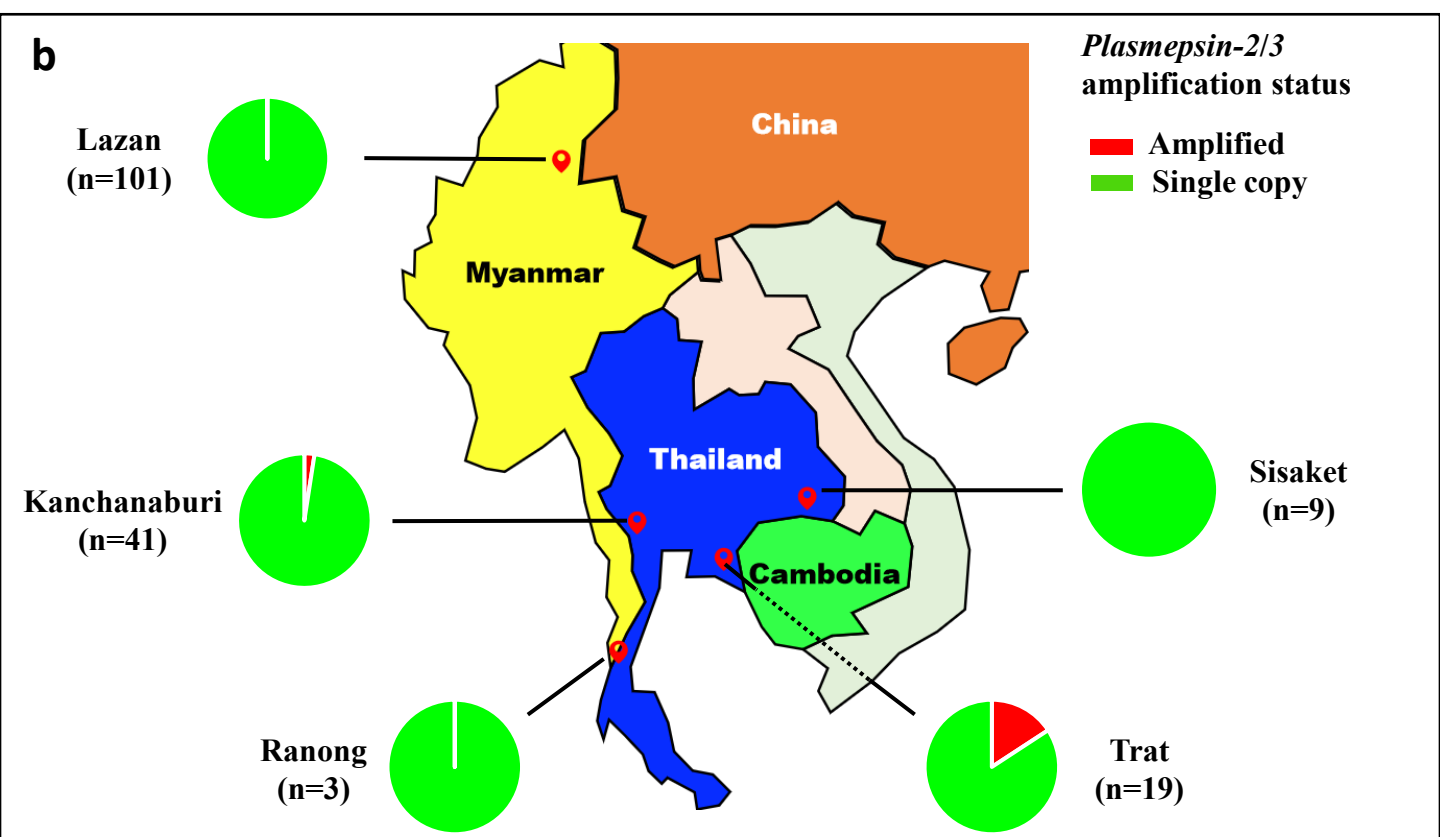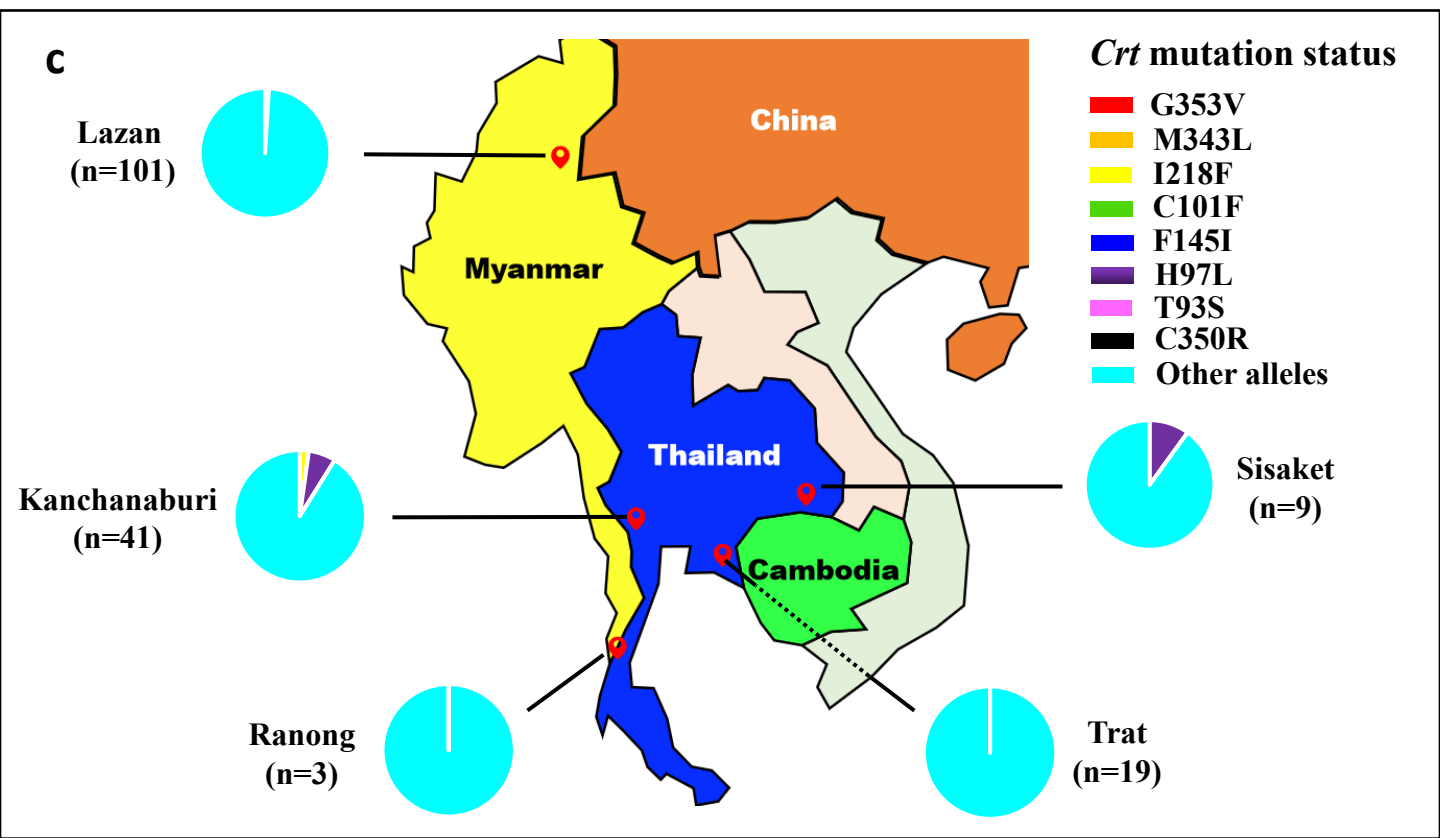

Supplement: Supplementary file 2 — Additional file 7: Figure S1. The K13 mutation status, plasmepsin 2/3 amplification status, and PfCRT mutation status by site and country. (a) KelchX mutation status indicates parasites with a K13 mutation other than C580Y and F446I. (b) “Single copy” indicates parasites without amplification of plasmepsin 2/3. (c) Other (PfCRT) alleles indicate parasites carrying no mutations at positions 93, 97, 145, 218, 343, 350, and 353 of the pfcrt gene. The shapefile map of Southwestern China, Cambodia, Myanmar, and other countries was downloaded and prepared by using Pixelmap Generator-Beta online (amCharts, Vilnius, Lithuania) (https://pixelmap.amcharts.com/), which is copyright free. [file 13071_2022_5239_MOESM2_ESM.pdf]

CM

TC

TM

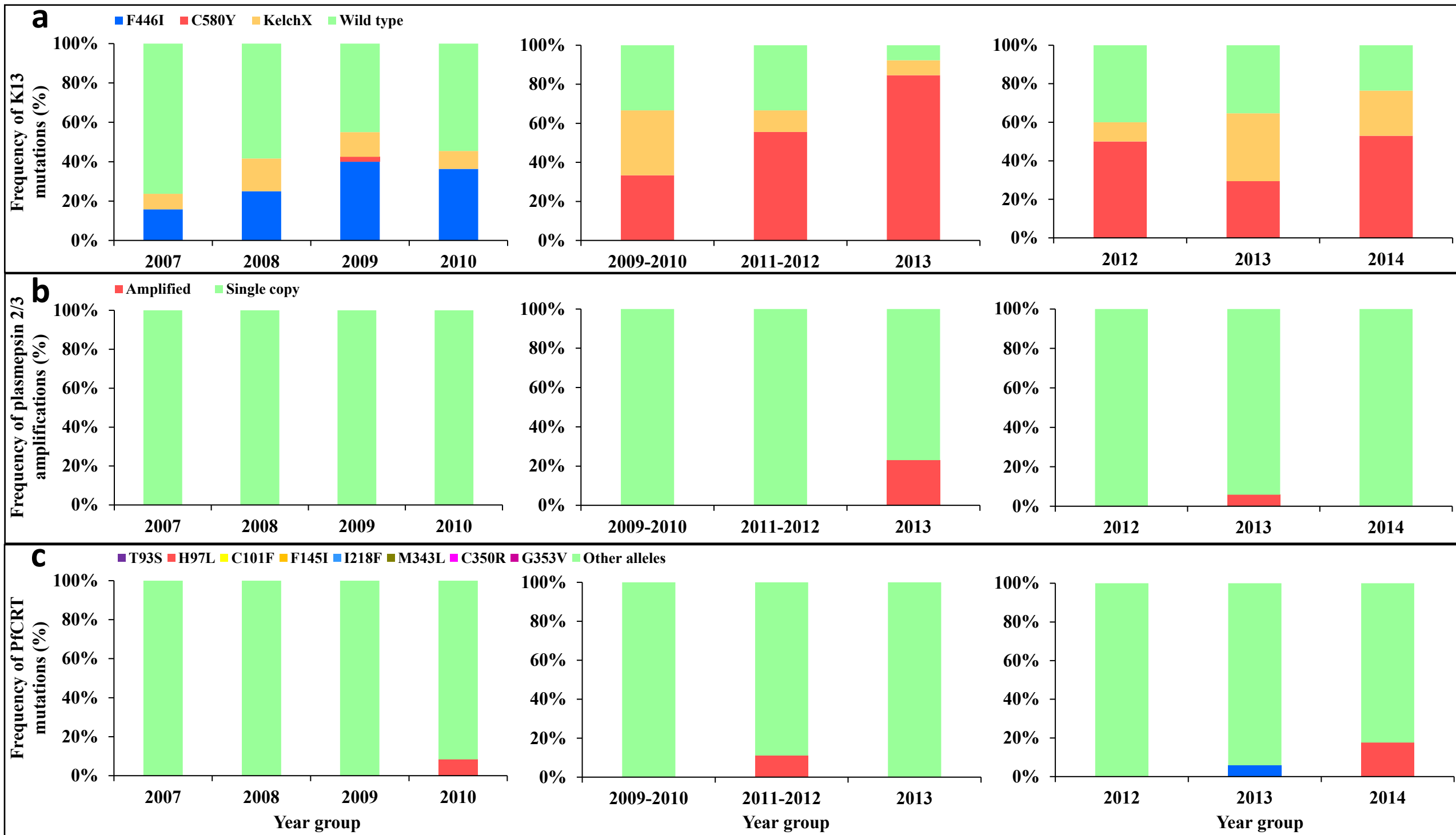

Supplement: Supplementary file 7 — Additional file 7: Figure S2. (a) Frequency of K13 mutations in CM, TC, and TM in different year groups. KelchX mutation status indicates parasites with a K13 mutation other than C580Y and F446I. (b) Frequency of plasmepsin 2/3 amplifications in CM, TC, and TM in different year groups. ”Single copy” indicates parasites without amplification of plasmepsin 2/3. (c) Frequency of PfCRT mutations in CM, TC, and TM in different year groups. ”Single copy” indicates parasites without amplification of plasmepsin 2/3. Other (PfCRT) alleles indicate parasites carrying no mutations at positions 93, 97, 145, 218, 343, 350, and 353 of the pfcrt gene. [file 13071_2022_5239_MOESM7_ESM.pdf]
