# Supplementary material for: Evaluations of candidate markers of dihydroartemisinin-piperaquine resistance in Plasmodium falciparum isolates from the China–Myanmar, Thailand–Myanmar, and Thailand–Cambodia borders
Source: Parasit Vectors. 2022 Apr 12;15:130. doi: 10.1186/s13071-022-05239-1 (PMC9004172; doi:10.1186/s13071-022-05239-1)
Supplement: Supplementary file 3 — Additional file 3: Table S2. The prevalence of K13 mutations, plasmepsin 2/3 CNVs, and PfCRT mutations in different border regions. Fisher’s exact test was done to assess the difference in the frequency of mutations or CNVs between borders using SPSS (version 21.0 for Windows). P < 0.05 was considered statistically significant. [file 13071_2022_5239_MOESM3_ESM.docx]

**Table S2. The prevalence of K13 mutations, *plasmepsin* 2/3 CNVs and PfCRT mutations in different border regions.**

| **Molecular Markers of Resistance** | **SNPs/CNV** | **Regions** | | | ***P* value** |
| --- | --- | --- | --- | --- | --- |
|  |  | **Thailand-Cambodia** | **Thailand-Myanmar** | **China-Myanmar** |  |
| **PfCRT mutations** | C72S | 0% (0) | 0% (0) | 0% (0) | / |
|  | M74I | 100% (28/28) | 100% (44/44) | 98.02% (99/101) | 0.701 |
|  | N75E/D | 100% (28/28) | 100% (44/44) | 98.02% (99/101) | 0.701 |
|  | **K76T** | **100% (28/28)** | **100% (44/44)** | **98.02% (99/101)** | **0.701** |
|  | A220S | 100% (28/28) | 100% (44/44) | 96.04% (97/101) | 0.288 |
|  | I356L | 92.86% (26/28) | 97.73% (43/44) | 94.06% (95/101) | 0.639 |
|  | T93S | 0% (0) | 0% (0) | 0% (0) | / |
|  | **H97L** | **3.57% (1/28)** | **6.82% (3/44)** | **1% (1/101)** | **0.081^#^** |
|  | C101F | 0% (0) | 0% (0) | 0% (0) | / |
|  | F145I | 0% (0) | 0% (0) | 0% (0) | / |
|  | I218F | 0% (0) | 2.27% (1/44) | 0% (0) | 0.416 |
|  | M343L | 0% (0) | 0% (0) | 0% (0) | / |
|  | C350R | 0% (0) | 0% (0) | 0% (0) | / |
|  | G353V | 0% (0) | 0% (0) | 0% (0) | / |
| **K13 Mutations^a^** | P441L | 0% (0) | 0% (0) | 1.98% (2/101) | 0.701 |
|  | P443S | 0% (0) | 0% (0) | 1% (1/101) | 1.000 |
|  | **F446I** | **0% (0)** | **0% (0)** | **28.71% (29/101)** | **0.000**^***^ |
|  | F483S | 0% (0) | 0% (0) | 1% (1/101) | 1.000 |
|  | L492S | 0% (0) | 0% (0) | 1% (1/101) | 1.000 |
|  | Y493H | 7.14% (2/28) | 0% (0) | 0% (0) | 0.025^*^ |
|  | F495S | 0% (0) | 0% (0) | 1% (1/101) | 1.000 |
|  | Y519K | 0% (0) | 0% (0) | 1% (1/101) | 1.000 |
|  | G538V | 0% (0) | 6.82% (3/44) | 0% (0) | 0.019^*^ |
|  | R539T | 7.14% (2/28) | 2.27% (1/44) | 1% (1/101) | 0.174 |
|  | E556D | 0% (0) | 0% (0) | 1% (1/101) | 1.000 |
|  | R561H | 0% (0) | 2.27% (1/44) | 0% (0) | 0.416 |
|  | P574L | 0% (0) | 9.09% (4/44) | 1% (1/101) | 0.020^*^ |
|  | **C580Y** | **64.29% (18/28)** | **43.18% (19/44)** | **1% (1/101)** | **0.000**^***^ |
|  | A675V | 0% (0) | 4.55% (2/44) | 1% (1/101) | 0.228 |
| ***plasmepsin* 2/3 amplification** | **CNVs of PM2/3** | **10.71% (3/28)** | **2.27% (1/44)** | **0% (0)** | **0.008**^**^ |

Note, ***, *P* < 0.001; **, *P* < 0.01; *, *P* < 0.05; 'a', analyzed based on data collected from our previous study [28].
